# Combining PARP inhibition and immune checkpoint blockade in ovarian cancer patients: a new perspective on the horizon?

**DOI:** 10.1016/j.esmoop.2022.100536

**Published:** 2022-07-15

**Authors:** L. Musacchio, C.M. Cicala, F. Camarda, V. Ghizzoni, E. Giudice, M.V. Carbone, C. Ricci, M.T. Perri, F. Tronconi, M. Gentile, V. Salutari, G. Scambia, D. Lorusso

**Affiliations:** 1 Department of Women and Child Health, Division of Gynecologic Oncology, Fondazione Policlinico Universitario Agostino Gemelli IRCCS, Rome, Italy; 2 Department of Medical and Surgical Science, Medical Oncology Unit, Comprehensive Cancer Center, Fondazione Policlinico Universitario Agostino Gemelli IRCCS, Rome, Italy; 3 Medical Oncology Unit, Marche Polytechnic University, Ancona, Italy; 4 Department of Biomedical Sciences and Human Oncology, University of Bari, Bari, Italy; 5 Department of Life Science and Public Health, Catholic University of Sacred Heart Largo Agostino Gemelli, Rome, Italy

**Keywords:** ovarian cancer, immunotherapy, immune checkpoint inhibitors, PARP inhibitors

## Abstract

Immune checkpoint inhibitors (ICIs) have completely reshaped the treatment of many malignancies, with remarkable improvements in survival outcomes. In ovarian cancer (OC), however, this emerging class of drugs has not yet found a favorable use due to results from phase I and II studies, which have not suggested a substantial antitumoral activity of these agents when administered as monotherapy. Robust preclinical data seem to suggest that the combination ICIs with poly(ADP-ribose) polymerase (PARP) inhibitors (PARPis) may result in a synergistic activity; furthermore, data from phase II clinical studies, evaluating this combination, have shown encouraging outcomes especially for those OC patients not suitable for platinum retreatment. While waiting for ongoing phase III clinical trial results, which will clarify the role of ICIs in combination with PARPis in the newly diagnosed OC, this review aims to summarize the preclinical data and clinical evidence available to date.

## Highlights

• Preclinical data indicate that PARPis exhibit immune modulating properties.
• The combination of PARPi with ICIs displays significant synergistic activity in preclinical models.
• Phase I and II clinical trials showed encouraging results for this combination, especially in platinum-resistant OC.
• Four ongoing phase III trials exploring the combination in first-line setting will delineate the role of immunotherapy in OC.

## Introduction

The introduction in the ovarian cancer (OC) therapeutic algorithm of poly(ADP-ribose) polymerase (PARP) inhibitors (PARPis) in both recurrent and first-line setting has notably improved survival outcomes of these patients.^1^^,^^2^ However, despite these unprecedent results with substantial improvement in clinical care, OC remains the most lethal among gynecological malignancies with an estimated 12 810 new deaths in 2022,^3^ thus highlighting a need for new strategies of treatment.

Over the past decade, monoclonal antibodies that block immune checkpoints, such as programmed cell-death 1 (PD-1) and cytotoxic T-lymphocyte antigen 4 (CTLA-4), have completely reshaped the treatment of multiple malignancies, demonstrating remarkable gain in progression-free survival (PFS) and overall survival (OS).^4^ The potential antitumor activity of immune checkpoint inhibitors (ICIs) is mainly based on the intrinsic characteristics of the tumor cells that include gene mutations resulting in abnormal protein expression patterns, such as neoantigens or tumor-associated antigens, and that lead to initiation of the immune system process with activation and infiltration of T cells, and recognition and killing of tumor cells.^5^^,^^6^

By contrast, chronic PARP inhibition leads to sustained DNA damage that promotes several cellular mechanisms, such as increasing genomic instability, immune pathway activation, and programmed death-ligand 1 (PD-L1) expression on cancer cells, which might promote responsiveness to ICIs.

In this review, we discuss the scientific rationale supporting the combined use of PARPis with ICIs in OC, exploring preclinical data and both published and ongoing trial.

## Background and preclinical data

Over the last few years, the potential role of PARPis in immune response activation against tumor cells has been intensively investigated in preclinical studies. Besides their main mechanism of action, which consists in the disruption of the DNA repair machinery in tumor cells harboring mutations in genes involved in this process (e.g. BRCA),^7^ a growing body of evidence has shed light on the immunomodulatory properties of PARP inhibition. This inhibition occurs by preventing the release of PARP-1/2 from chromatin, impairing the DNA repair machinery. PARPis show different ‘PARP-trapping’ activity based on their molecular structure; currently approved olaparib, niraparib, and rucaparib have a 100-fold greater PARP-trapping activity than the investigational agent veliparib.^8^ Preclinical studies suggest that PARPis may enhance the immune response to tumor cells not only by activating an innate response through the release of tumor neoantigens and danger-associated molecular patterns, but also by stimulating an adaptive immune response and by reshaping of the tumor microenvironment.^9^

### Neoantigen formation and immunogenic cell death

One of the key mechanisms that explains the immunogenic potential of PARP inhibition is the generation of neoantigens through the alteration of the DNA repair in tumor cells. Neoantigens are neoepitopes that may develop as a result of the high rate of somatic mutations that occur in tumor cells; being entirely tumor specific, immune cell (IC) response to neoantigens is of particular interest because it is not affected by central T-cell tolerance and it is not expected to result in autoimmune toxicity, in contrast to T-cell response to self-antigens.^6^^,^^10^ Highly mutated tumors, for example, those identified by a high tumor mutational burden, exhibit a strong immunogenic phenotype, which results in an important and durable response to immunotherapy.^11^ In addition, tumor harboring deficiencies in genes involved in DNA damage repair show remarkable responses to ICIs. This relationship between the disruption of DNA damage response and ICIs has been extensively studied in cancers harboring defects in mismatch repair genes^12^; new potential candidate biomarkers of immune response are under investigation such as CDK12 mutations, which have been described as a feature of immune responsiveness in other solid neoplasms.^13^ Taken together, these observations imply that the inactivation of the DNA repair machinery results in an increased rate of mutations and, consequently, in an increased production of tumor neoantigens, which could trigger immune detection.^14^ In this context, by affecting the homologous recombination (HR) pathway in tumor cells, PARPis may enhance the formation of neoantigens and may foster the recognition of tumor cells by the immune system.

Along with the generation of a T-cell response, the accumulation of unrepaired double-strand breaks induced by PARPis results in immunogenic cell death, which in turn leads to the release of danger-associated molecular patterns. These products of tumor cell death promote the recruitment of antigen-presenting cells at tumor site, thus eliciting an immune response.^15^

### Activation of the stimulator of interferon genes (STING) pathway

In addition to the increased mutational rate and the production of neoantigens, new evidence suggests that PARPis may regulate immune response through a tumor-cell-intrinsic pathway. Damaged cytosolic DNA can induct an innate immune response through the cyclic GMP–AMP synthase/stimulator of interferon genes (cGAS/STING) pathway; cGAS acts as a sensor of cytosolic DNA, and its binding activates the catalytic subunit of this enzyme, which leads to the activation of STING.^16^

In OC cell lines treated with a PARPi, it has been shown that accumulation of cytosolic double-stranded DNA induces, via cGAS, the activation of the STING pathway, through the increased phosphorylation and nuclear translocation of the transcriptional regulatory factors TANK-binding kinase 1 (TBK1) and interferon regulatory factor 3 (IRF3). Downstream targets of STING pathway include CCL5 and CXCL10, two molecules implicated in T-cell recruitment; the *in vivo* model showed that PARPi-treated tumors display significantly higher percentage of CD8+ cells and programmed death-ligand 1 (PD-L1)-positive cells; moreover, this effect was not observed in an immunodeficient mice model, thus indicating that the antitumor effect of PARPis requires a functional immune system.^17^

### Increased PD-L1 expression and immune cell infiltration

Another mechanism linked to the immunogenic potential of PARPis is the increased expression of PD-L1 induced by these drugs. However, the consequences of this process are not completely univocal. *In vitro* and *in vivo* models have demonstrated that PARP inhibition enhances PD-L1 expression through the increased phosphorylation of CHK2,^18^ a kinase involved in the response to DNA damage, and through the inactivation of GSK3β, which negatively regulates PD-L1 expression by proteasome-dependent degradation.^19^ Downregulation of BRCA did not significantly affect the expression of PD-L1. In the model by Jiao et al.,^18^ PARPi-induced PD-L1 upregulation attenuates tumor immune response; PD-L1 pharmacological blockade could restore T-cell killing. In OC cell lines, olaparib determined increased expression of PD-L1 on tumor cells in a dose-dependent manner. This effect was not observed in BRCA-mutated cell lines; however, *in vivo*, the effect of combined treatment on PD-L1 expression and tumor growth was observed in both BRCA-mutated (BRCAmut) and BRCA-wild-type (BRCAwt) transplanted tumor tissue.^20^ In combination, these results imply that the mechanisms of increased PD-L1 expression induced by PARPi are extremely complex and may involve multiple transduction pathways.^21^

Of note, the immune responses observed in both the *in vitro* and *in vivo* models were independent of the BRCA1/2 mutational status. Mutations in BRCA1 and BRCA2 are known to be synthetic lethal with PARP inhibition^22^; therefore, treatment of BRCA-mutated tumors has been the backbone of the clinical development of PARPi. It is unclear whether the BRCA mutational status may contribute to the immunomodulatory effect of PARP inhibition. In a BRCA1-deficient mouse model of OC,^23^ treatment with olaparib resulted in a significant increase of intratumoral effector CD4+ and CD8+ cells along with a reduced expression of the co-inhibitory receptors PD-1/Tim-3 and PD-1/Lag-3 on CD8+ cells. The addition of a PD-1 antibody to the treatment with olaparib alone resulted in a prolonged OS compared with olaparib monotherapy; this could indicate that resistance to olaparib may be driven by the activation of immune-suppressing pathways that could be counteracted by the addition of an ICI. Of note, neither an increase of intratumoral effector T cells nor a clinical effect on tumor progression was observed in the BRCA1-proficient model, in contrast to the increased immune response observed in OC cell lines by Shen et al.,^17^ which was independent by the BRCA mutational status.

The full knowledge of all the possible interactions between PARP inhibition and the immune response remains far from understood. For example, the chemokine CCL5 has been described also as a driver of myeloid-derived suppressor cells,^24^ a subpopulation of myeloid cells that may counteract the immune response to the tumor.^25^ In addition, enhanced expression of PD-L1 may lead to the activation of an immune checkpoint blockade which may antagonize the immunogenic response induced by PARP inhibition. In the murine model by Shen et al.,^17^ combined treatment with a PARPi and an anti PD-L1 antibody resulted in a synergistic effect; moreover, compared with both PARPi and anti-PD-L1 monotherapy, the combination produced the most significant increase in CD8+ cells.

### Modification of tumor microenvironment

The role of PARP-1/2 in DNA damage repair, cell cycle regulation, chromosome function, genome stability, and angiogenesis is well established.^26^^,^^27^ The impact of PARP-1 and PARP-2 on immune homeostasis is more controversial. Evidence derived from preclinical studies showed a role of PARP-1 and PARP-2 in T- and B-cell development, confirmed by the presence of lymphopenia in mice with dual PARP-1/2 deficiency likely caused by an accumulation of unrepaired DNA damages during IC proliferation.^28^^,^^29^ Concerning T cells, PARP-1 plays several roles in CD4+ cell differentiation. A PARP-1 deficiency leads to an increase of regulatory T cells (Tregs). The differentiation of T cells from CD4+ T cells to Tregs is mediated by a transcriptional factor, the forkhead box p3 factor (Foxp3). PARP-1 regulates Foxp3 expression through a post-transcriptional PARylation causing its degradation.^30^ An increase in Tregs is usually associated with an immunosuppressive status especially in peripheral blood, although the release of interleukin-10 mediated by Tregs represents an immunogenic signal in several contexts.^31^ Furthermore, PARP-1 deficiency leads to a downstream T-cell activation mediated by the nuclear factor of activated T cells (NFAT)^32^ but at the same time promotes differentiation of CD4+ cell to a Th1 proinflammatory phenotype.^30^^,^^33^ PARP-1 and PARP-2 have also demonstrated an impact on innate immune system cells with several mechanisms such as the modulation of natural killer cells, dendritic cells, macrophages, and recruitment of neutrophils.^34^

The complex relationship between cancer cells and tumor microenvironment, composed by fibroblast, endothelial cells, resident and peripheral ICs, has a crucial role during tumorigenesis and influences the drug response. Tumor microenvironment cells also concur to anti-cancer immune escape through the expression of inhibitor receptors on cancer and immune surface, such as the programmed death-ligand 1 and 2 (PD-L1, PD-L2) and the CTLA-4. For this purpose, several drugs have been approved to reactivate immune response directed against these markers (anti-PD-L1, anti-PD-1, anti-CTLA4). Along with the drugs specifically targeting these receptors, there is evidence that other agents, such as the PARPi, may reinforce the immune response by unbalancing this environment in favor of an immunogenic status. The PARPi-promoted upregulation of apoptosis receptor and the sensitization of TNF-related apoptosis-inducing ligand (TRAIL) promote natural killer cell activation.^35^^,^^36^ Moreover, the aforementioned activation of STING pathway induced by PARPi leads to the expression of INF-1 and T-cell-recruiting chemokines, especially in BRCA-mutated cells.^37^ This effect has been confirmed in preclinical models demonstrating an increase of the CD4+ and CD8+ population in BRCA1-mutated OC cells treated with olaparib.^23^

The association of niraparib (PARPi) and an anti-PD-1 was evaluated *in vitro* and *in vivo* from samples, both human and murine, of high-grade serous OC.^38^ The study confirmed the increase of PD-L1 expression after niraparib treatment in both experimental models. The murine models treated with the addition of anti-PD-L1 showed a greater decrease in tumor growth without a significantly higher toxicity. To better understand the mechanisms below this response, a coculture of high-grade serous OC cells and peripheral blood CD8+ cells from healthy donors was carried out. The addition of niraparib and the anti-PD-L1 was associated with an increase of cytokines level, underlying an activation of CD8+ cells against cancer cells. Moreover, the murine models treated with combination therapy showed a higher proportion of CD4+ and CD8+ T cells in peripheral blood.

### Rationale for the combination of PARPi + ICI

Given the immunological properties of PARPi, the combination of these drugs with ICIs may be an intriguing strategy to enhance immune response to OC cells. The first evidence of a synergism between ICIs and PARPi was observed in a murine BRCA-deficient OC model treated with veliparib in combination with an anti-CTLA-4 antibody or an anti-PD1/PD-L1 antibody. Of note, in this model, while the inhibition of CTLA-4 resulted in an increased T-cell recruitment and activation and in significantly longer survival, PD-1/PD-L1 blockade did not determine such immune activation and did not result in improved survival.^39^ However, subsequent research studies shed light on the existence of a synergism between PARPi and PD-1/PD-L1 inhibitors. In a preclinical murine model, the PARPi niraparib increased the activity of interferon-activated pathways and the infiltration of T cells in tumors. Combination of PARPi and an anti-PD-1 antibody demonstrated synergistic activity in a BRCA-deficient model of OC. This is in line with the work by Ding et al.,^23^ in which the addition of a PD-L1 antibody to olaparib treatment resulted in an increased therapeutic effect. Of note, in the work by Wang et al.,^40^ the synergism between PARPi and anti-PD-1/PD-L1 ICI was observed also in different BRCA-proficient murine cancer models. More recently, in a three-dimensional model of OC spheroid culture, patient-specific tumor cells and ICs were incorporated into 3D spheroids and analyzed for modifications in IC recruitment and activation. It has been shown that treatment with an anti-PD-1 alone (pembrolizumab) did not result in a significantly reduced spheroid viability, while olaparib monotherapy showed some efficacy in one of the two spheroid models. Nevertheless, combination treatment with anti PD-1/PD-L1 and olaparib led to a markedly reduced spheroid viability in both models.^41^

Immunogenomic profiling of the TOPACIO trial of niraparib + pembrolizumab in OC identified two main determinants of response to the combination: one is the so-called mutational signature 3, which is associated with HR deficiency (HRD) and was predictive of longer PFS; and the other is a positive immune score, defined by interferon pathways activation or by the percentage of interferon-primed exhausted CD8+ cells. These results further underline the link between the deficiency in DNA damage repair and tumor immunogenicity.^42^

## Published trials with the combination of parpi and icis

Several clinical phase I/II studies investigated the combination of PARPi and ICIs (Table 1).

**Table 1:** Summary of published trials providing information on drugs, inclusion setting criteria, number of patients, efficacy, and survival results

	Konstantinopoulos et al.^43^	Drew et al.^48^, ^49^	Zimmer et al.^50^	Lee et al.^51^	Liu et al.^52^	Freyer et al.^53^
Phase	I–II	I–II	I	II	II	II
Drug	Pembrolizumab + niraparib	Durvalumab + olaparib ± bevacizumab	Durvalumab + olaparib ± cediranib	Durvalumab + olaparib	Dostarlimab + niraparib + bevacizumab	Durvalumab + olaparib + bevacizumab
Setting	Patients with advanced or metastatic triple-negative breast cancer or recurrent ovarian cancer (KEYNOTE-162)	Patients with gBRCAwt ROC and had progressed after receiving one or two prior lines of platinum-based chemotherapy (MEDIOLA)	Patients with a diagnosis of non-small-cell lung cancer, TNBC, ovarian cancer, and metastatic castrate-resistant prostate cancer	Patients with ROC who have received at least two prior regimens or who are platinum resistant or refractory during or after a first platinum containing regimen (OvCA)	Patients with platinum-resistant epithelial ROC or recurrent carcinosarcoma of the ovary, after one or two lines of standard chemotherapy (OPAL)	Patients with platinum-resistant and platinum-sensitive ROC, regardless the number of previous lines of therapy (GINECO BOLD)
Patients enrolled (*n*)	62 (ROC)	32 (doublet) + 31 (triplet)	Nine patients with ovarian cancer, primary peritoneal cancer, endometrial carcinoma, and TNBC received triplet therapy	35	41	74 (41 in the platinum-resistant cohort; + 33 in the platinum-sensitive cohort)
Median age (range), years	60 (46-83)	68.5 (doublet) + 64.0 (triplet)	59 (44-73)	67 (40-85)	66 (37-83)	66 (38-89) (PlR) 65 (49-81) (PlS)
PFS months (95% CI)	3.4 (2.1-5.1)	5.5 (3.6-7.5) versus 14.7 (10.0-18.1)	—	—	7.6 (4.2-10.6)	4.1 (3.5-5.9) (PlR)^a^ 4.9 (2.9-7.0) (PlS)^a^
OS months (95% CI)	—	—	—	—	—	18.8 (9.6-NR) (PlR)^a^ 18.5 (15.6-NR) (PlS)^a^
ORR (%)	18	34.4 versus 87.1	44	15	17.9	—
Best response	CR	CR	PR	PR	PR	—
**DOR**	NR in patients with CR or PR	6.9 versus 11.1	8.5 (7-26)	—	—	—

CI, confidence interval; CR, complete response; DOR, duration of response; gBRCAwt, germinal BRCA wild type; NR, not reached; ORR, overall response rate; OS, overall survival; PFS, progression-free survival; PR, partial response; ROC, recurrent ovarian cancer; TNBC, triple-negative breast cancer.

a Calculated as 90% CI.

The TOPACIO/KEYNOTE-162 (NCT02657889) was an open-label single-arm phase I–II trial designed to evaluate the association of niraparib and pembrolizumab in triple-negative breast cancer and recurrent OC (ROC), regardless of BRCA status.^43^ Phase I study had as primary endpoint the number of patients reporting dose-limiting toxicities, while phase II trial evaluated the objective response rate (ORR). Secondary endpoints included PFS, disease control rate (DCR), duration of response (DOR), OS, safety, and the pharmacokinetics (PK) of niraparib and the major metabolite of combination therapy. The exploratory endpoint investigated the correlation between BRCA and HRD status with the treatment efficacy.

The recommended phase II dose (RP2D) of niraparib was fixed at 200 mg daily. A total of 53 patients with OC were enrolled in the phase II study and treated with niraparib + pembrolizumab 200 mg every 21 days. As many as 11 patients had BRCA 1-2 mutations (18%) at data cut-off, 49 patients had a progression disease (41 radiological and 8 clinical), in 5 patients the treatment was interrupted for adverse events, and 5 patients withdrew from the study. The ORR was 18% [90% confidence interval (CI) 11% to 29%], with three complete response (CR) and eight partial responses (PRs). The DCR was 65% (90% CI 54% to 75%) and the median DOR had not been reached at data cut-off in patients with a CR or a PR. The median PFS was 3.4 months (95% CI 2.1-5.1 months); the OS data were still not mature. No correlation with BRCA and HRD status, PD-L1 expression, and prior bevacizumab treatment was observed. The most common treatment-related adverse events of at least grade 3 were anemia (11, 21%) and thrombocytopenia (5, 9%). Although the trial did not meet the predefined endpoint, the combination of niraparib and pembrolizumab showed a meaningful activity compared with single-agent-based therapy in the same population of patients with resistant or refractory OC.^44^, ^45^, ^46^, ^47^

The MEDIOLA (NCT02734004) study was an open-label phase I/II basket trial including four cohorts of PARPi and ICIs-naïve patients. Each cohort was treated with olaparib 300 mg twice daily in combination with durvalumab 1.5 g intravenously every 4 weeks, after a 4-week run-in with olaparib alone; the treatment was continued until disease progression. The primary endpoints were DCR after 12 weeks and the safety. Secondary endpoints included DCR evaluated after 28 weeks, DOR, ORR, PFS, OS, and PK parameters.

The OC cohort included 32 patients with platinum-sensitive ROC with a germline BRCA1/2 mutation and previously treated with at least one platinum-based chemotherapy (ChT).^48^ The DCR after 12 weeks was 81% (44% of PR and 19% of CR), at 28 weeks 65.6% (90% CI 49.6% to 79.4%). The ORR was 71.9% (95% CI 53.25% to 86.25%). The most common grade ≥3 adverse events were anemia (9%), increased lipase (9%), increased amylase (6%), and neutropenia (3%).

A second-stage phase II study was designed to test the addition of bevacizumab 10 mg/kg intravenously every 2 weeks to olaparib and durvalumab, in germline BRCAwt relapsed OC patients.^49^ Primary endpoints were DCR at 24 weeks and safety. Secondary endpoints included DCR at 56 weeks, ORR, DOR, PFS, OS, and PK.

The triplet demonstrated a superiority over the doublet for all the endpoints, with a 24-week DCR of 77.4% (90% CI 61.7-88.9) versus 28.1% (90% CI 15.5-43.9). respectively. The ORR was 87.1% (95% CI 70.2-96.4) versus 34.4% (95% CI 18.6-53.2). The median PFS was 14.7 months (95% CI 10.0-18.1). The adverse events reported were consistent with those previously reported for the single drugs. Interestingly, at a following exploratory analysis, genomic instability did not correlate with response, which was confirmed to be ≥75% in all subgroups.

Zimmer et al.^50^ designed another multidrug study (NCT02484404) with durvalumab associated with olaparib and/or an anti-VEGFR1-3, cediranib, involving a phase I stage including several solid tumors (non-small-cell lung cancer, triple-negative breast cancer, OC, and metastatic castrate-resistant prostate cancer) and a phase II stage addressed exclusively to the patients with ROC.

Phase I primary endpoint was the determination of the RP2Ds. Secondary endpoints included safety, efficacy (ORR, PFS, OS, DOR), and PK. Eligible patients, during phase I trial, received a dose escalation of all three drugs. The starting doses were durvalumab 1500 mg every 28 days, olaparib 300 mg twice daily, and cediranib 15 mg once daily in a week schedule consisting in 5 days on and 2 days off. The dose level 2 provided only an increase of cediranib up to 20 mg once daily. Among patients with gynecological neoplasms, six had OC, of which five had a resistant disease. Seven of nine patients had a germinal BRCAwt (gBRCAwt) tumor.

The RP2D was defined as the dose level 2. The most common grade 3 adverse event was lymphopenia. The ORR was 44% (4 PR and 0 CR) with a median DOR of 8.5 months (range 7-26 months). A correlation between PD-L1 positivity and ORR was observed. All the seven patients with PD-L1-positive cancer cells had a PR or a stable disease (SD), while the two PD-L1-negative patients had progressive disease.

The results of a phase II stage, which evaluated the combination of olaparib and the anti-PD-1 durvalumab in women with ROC, were presented at ESMO Congress in 2018.^51^ The majority of the patients included (*n* = 30, 86%) had a diagnosis of platinum-resistant OC. Patients were enrolled regardless of BRCA status. A total of 35 patients were enrolled and among the 34 evaluable, 5 achieved a PR [2 with gBRCA mutated (gBRCAm)] and 13 had an SD (3 gBRCAm/10 BRCAwt). Most frequent adverse grade 3/4 events were anemia (26%) and lymphopenia (14%). The study is still recruiting; final results are awaited to draw definitive conclusions.

During the SGO 2021 meeting, the results of the OPAL trial (NCT03574779) were presented. This was a phase II multicohort study designed to evaluate the efficacy and the safety of niraparib with dostarlimab and bevacizumab in patients with platinum-resistant EOC, or ovarian carcinosarcoma.^52^ The trial was designed as a single-arm study with dostarlimab 500 mg IV every 3 weeks for the first four doses and then 1000 mg every 6 weeks, bevacizumab 15 mg/kg every IV 3 weeks up to 15 months, and niraparib 300 or 200 mg (according to weight <77 kg or platelet count at screening) orally once daily. The treatment continued until PD or unacceptable toxicity. Thirty-nine patients were evaluated for ORR (primary endpoint) showing seven PR and any CR (ORR 17.9%; 95% CI 8.7-31.1). At subgroup analysis, the ORR was consistent across the subgroups, although the patients previously treated with bevacizumab showed a lower response rate. Nearly 78.0% of patients reported at least grade ≥3 adverse events related to treatment, of which the most common were hypertension (22%) and thrombocytopenia (22%). The authors underlined that most patients enrolled had BRCAwt (34/41) or HRD-negative tumors (31/41). These factors, usually associated with lower responses to therapy, did not compromise the response rate.

In addition, results of the open-label, single-arm phase II GINECO BOLD study were presented at the ESMO 2021 meeting. This study was designed to assess the efficacy and toxicity of durvalumab, bevacizumab, and olaparib combination in OC patients with platinum-sensitive and platinum-resistant recurrence. DCR at 3 and 6 months was the primary endpoint. The study included 74 patients: 41 with platinum-sensitive recurrence and 33 with platinum-resistant relapse. With a median follow-up of 15.5 months, in the platinum-resistant relapse cohort DCR was 70% (90% CI 56-80) at 3 months and 44% (90% CI 29-57) at 6 months in the platinum-sensitive relapsed cohort. Median PFS in platinum-resistant relapsed and platinum-sensitive relapsed patients were 4.1 (90% CI 3.5-5.9) and 4.9 (90% CI 2.9-7) months, respectively. Median OS were 18.8 [90% CI 9.6-not reached (NR)] and 18.5 (90% CI 15.6-NR) months. Neither toxic deaths nor major safety signal were observed.^53^

## Ongoing clinical trials

The combination between PARPis and ICIs is currently being evaluated in multiple clinical trials, both in first-line setting and in pretreated patients (Table 2).

**Table 2:** Summary of ongoing trials providing information on primary endpoints, estimated study’s end date, and the clinical trial identifier.

Phase	Description	Agents	Primary endpoints	Estimated completion date	Trial Identifier
II	A Pilot Study to Evaluate the Efficacy and Safety of Preoperative Olaparib Monotherapy and Preoperative Olaparib Plus Pembrolizumab Combination Therapy in Patients With HRD-Positive Stage III or IV Advanced Epithelial Ovarian/Fallopian Tube/Primary Peritoneal Cancer^56^	Pembrolizumab and olaparib	Objective response rate	December 2023	NCT04417192
II	An uMbrella Study of BIomarker-driven Targeted Therapy In Patients With Platinum-resistant Recurrent OvariaN Cancer (AMBITION)^59^	Durvalumab and olaparib	Objective response rate	September 2022	NCT03699449
II	A Randomized Clinical Trial Investigating Olaparib, Durvalumab (MEDI4736) and UV1 as Maintenance Therapy in BRCAwt Patients With Recurrent Ovarian Cancer^60^	Durvalumab, olaparib and UV1 vaccine	Progression-free survival	June 2026	NCT04742075
III	A Randomized Phase 3, Double-Blind Study of Chemotherapy With or Without Pembrolizumab Followed by Maintenance With Olaparib or Placebo for the First-Line Treatment of BRCA Non-mutated Advanced Epithelial Ovarian Cancer (EOC) (KEYLYNK-001/ENGOT-ov43/GOG-3036)^51^	Pembrolizumab and olaparib	Progression-free survival	May 2025	NCT03740165
III	A Phase III Randomised, Double-Blind, Placebo-Controlled, Multicentre Study of Durvalumab in Combination With Chemotherapy and Bevacizumab, Followed by Maintenance Durvalumab, Bevacizumab and Olaparib in Newly Diagnosed Advanced Ovarian Cancer Patients (DUO-O)^50^	Durvalumab and olaparib	Progression-free survival	December 2027	NCT03737643
III	ATHENA (A Multicenter, Randomized, Double-Blind, Placebo- Controlled Phase 3 Study in Ovarian Cancer Patients Evaluating Rucaparib and Nivolumab as Maintenance Treatment Following Response to Front-Line Platinum-Based Chemotherapy)^55^	Nivolumab and rucaparib	Progression-free survival	December 2030	NCT03522246
III	ENGOT-0V44 The FIRST (First-line Ovarian Cancer Treatment With Niraparib Plus TSR-042) Study: A Randomized, Double-blind, Phase 3 Comparison of Platinum-based Therapy With TSR-042 and Niraparib Versus Standard of Care Platinum-based Therapy as First-line Treatment of Stage III or IV Nonmucinous Epithelial Ovarian Cancer^54^	Dostarlimab and niraparib	Progression-free survival	June 2026	NCT03602859
III	Randomized Phase III Trial on NIraparib-TSR-042 (Dostarlimab) versus Physician's Choice CHEmotherapy in Recurrent, Ovarian, Fallopian Tube or Primary Peritoneal Cancer Patients Not Candidate for Platinum Retreatment: NItCHE Trial (MITO 33)^58^	Dostarlimab and niraparib	Overall survival	January 2025	NCT04679064
III	A Phase III Randomized, Double-blinded Trial of Platinum-based Chemotherapy With or Without Atezolizumab Followed by Niraparib Maintenance With or Without Atezolizumab in Patients With Recurrent Ovarian, Tubal or Peritoneal Cancer and Platinum Treatment-free Interval (TFIp) >6 Months^57^	Atezolizumab and niraparib	Progression-free survival	January 2025	NCT03598270

The double-blind, randomized, phase III DUO-O trial (NCT03737643) enrolls patients with newly diagnosed advanced OC, who are treated according to their tumor BRCA mutation (tBRCAm) status. Patients in the non-tBRCAm cohort are randomized 1:1:1 to receive ChT + bevacizumab followed by bevacizumab maintenance or first-line ChT + bevacizumab + the anti-PD-L1 durvalumab followed by maintenance with bevacizumab + durvalumab or the same first-line regimen followed by maintenance with bevacizumab + durvalumab + olaparib. Patients in the open-label tBRCAm cohort are treated with ChT (with optional use of bevacizumab) + durvalumab followed by maintenance with durvalumab + olaparib. Primary endpoint is PFS; key secondary endpoints include OS and ORR.^54^

In the phase III ENGOT-Ov43/KEYLYNK-001 (NCT03740165) trial, patients with BRCAwt advanced OC are randomized to receive pembrolizumab or placebo in association with standard upfront ChT followed by maintenance with pembrolizumab, with or without olaparib, or placebo. Primary endpoints are PFS and OS.^55^

With a similar study design, the phase III study ENGOT-Ov44/FIRST trial (NCT03602859) evaluates the combination of first-line standard of care (SoC) ChT (with the optional use of bevacizumab) followed by niraparib maintenance versus the combination of SoC and the anti-PD-1 antibody dostarlimab followed by maintenance therapy with niraparib and dostarlimab. This study is enrolling both BRCAmut and BRCAwt patients. Planned study design also included a third arm of SoC followed by placebo; however, in view of the positive results of the PRIMA and PAOLA-1 trials,^56^^,^^57^ this arm was discontinued to offer SoC treatment to all randomized patients. Primary endpoints of the FIRST trial are the investigator-assessed PFS in the intention-to-treat population and in the PD-L1-positive patients.^58^

Maintenance therapy after SoC ChT with the combination of rucaparib and nivolumab is being investigated in the phase III ATHENA trial (NCT03522246). In this study, patients with response to first-line ChT are randomized 4:4:1:1 to maintenance therapy with rucaparib + nivolumab (arm A); rucaparib + intravenous placebo (arm B); nivolumab + oral placebo (arm C); placebo combination (arm D). The trial will evaluate, as primary endpoint, the investigator-assessed PFS in two independent comparisons: in ATHENA-MONO, rucaparib monotherapy will be compared with placebo, while in ATHENA-COMBO, rucaparib monotherapy will be evaluated against the combination of rucaparib and nivolumab.^59^

The upfront combination of a PARPi and an ICI, within a ChT-free regimen, may represent an intriguing strategy for biomarker-selected patients. The OLAPEM trial (NCT04417192) is a phase II study evaluating the efficacy and the safety of preoperative olaparib monotherapy, with or without pembrolizumab, in untreated patients with HRD-positive tumors who are candidates for debulking surgery.^60^

Two phase III randomized trials are assessing the efficacy of the combination between PARPi and ICI in pretreated patients. The ENGOT-Ov41/ANITA study (NCT03598270) enrolls patients with ROC who are candidates for a platinum retreatment (e.g. with a platinum-free interval >6 months). In this double-blind, randomized trial, patients are randomized to receive SoC platinum ChT followed by niraparib maintenance or platinum ChT + atezolizumab (an anti-PD-L1) followed by maintenance niraparib + atezolizumab. Primary endpoint is PFS.^61^

The NITCHE/MITO 33 trial (NCT04679064) randomizes ROC patients not suitable for platinum retreatment to receive ChT or the combination of niraparib and dostarlimab. This trial allows previous therapy with a PARPi or an ICI. Primary endpoint of the study is OS.^62^

The combination of the olaparib with durvalumab in the setting of platinum-resistant ROC is also under evaluation in one arm of the AMBITION trial (NCT03699449), which is an umbrella study of biomarker-driven target therapy. More specifically, HRD-positive patients are randomized to receive olaparib + durvalumab or + the tyrosine kinase inhibitor cediranib. The primary endpoint is the ORR.^63^

Other immune-stimulatory agents are currently under study to investigate potential synergisms with PARPi. In the open-label three-arm phase II DOVACC trial (NCT04742075), patients with relapsed OC who have received at least two lines of platinum-containing ChT with response to the last course are randomized to receive maintenance therapy with olaparib alone, olaparib and durvalumab, or the combination of both drugs with UV1, a cancer vaccine targeting the universal tumor antigen telomerase. The study randomized its first patients in December 2021 and will have PFS as primary endpoint.^64^

The NCT03695380 trial investigates if the blockade of other signaling pathways may potentiate the effect of the combination of a PARPi and an ICI. Specifically, in this phase I trial, the oral MEK-inhibitor cobimetinib is administered with niraparib alone or in combination with atezolizumab. Endpoint of this trial is the safety of the combination; if the safety endpoint is met, enrollment in the randomized phase 2 study will begin.^65^

## Discussion

Although immunotherapy has dramatically revolutionized cancer therapy with outstanding achievements in many different malignancies, its results in OC have been disappointing so far. Single-agent ICI does not appear to provide a meaningful clinical benefit in patients with ROC or advanced OC, as demonstrated by the results of the KEYNOTE-100 trial. In this phase II study, which recruited previously treated OC patients with a platinum-free interval of at least 3 months, single-agent pembrolizumab yielded an ORR <10%, which was one of the primary endpoints of the study. The response rate was slightly higher, however <20%, in patients with a PD-L1 expression ≥10, measured by means of combined positive score.^47^ In addition, ICIs alone or in combination with ChT failed to show superiority over ChT alone in two phase III trials. In the JAVELIN Ovarian 200 trail, neither avelumab alone nor the combination of avelumab and pegylated liposomal doxorubicin (PLD) showed a survival gain over PLD alone in a population of patients with platinum-resistant or refractory OC.^66^ Likewise, in the NINJA trial, nivolumab did not improve OS and showed worse PFS compared with gemcitabine and PLD in the control arm, in patients with platinum-resistant OC.^67^

The activity of avelumab has also been tested in combination with ChT in the upfront setting. In the JAVELIN Ovarian 100 trial, patients with stage III-IV OC were randomized to receive front-line ChT with or without avelumab followed by avelumab maintenance or observation. However, at the interim analysis, avelumab did not show advantage in PFS nor in OS over standard treatment and the trial was thereupon halted, due to the crossing of prespecified futility boundaries, and is no longer enrolling.^68^

The lower response rate to ICIs and the absence of a survival benefit in these patients may derive from the immunological phenotype of OC, which is characterized by a low PD-L1 expression and a low tumor mutational burden that may promote neoantigen formation.^69^^,^^70^ In a phase III trial evaluating the addition of ICIs to first-line ChT, the proportion of patients with a PD-L1 score on ICs ≥5% was 20%. The IMagyn050 trial was a double-blind phase III trial in which patients were randomly assigned to atezolizumab or placebo in combination with ChT + bevacizumab. There was no PFS improvement for the combination of atezolizumab with bevacizumab and ChT in the intention-to-treat population, nor in the PD-L1-positive tumors (defined as IC ≥1%). The results of this trial demonstrate a lack of a survival benefit for the combination of ICIs and antiangiogenic drugs in OC,^69^ but in an exploratory subgroup analysis, a benefit was observed in patients with PD-L1 IC ≥5%. These results are encouraging but difficult to interpret, as this population represents only 6%. Anyway, this intriguing signal may warrant further evaluation of atezolizumab in a population with high PD-L1 expression.

Given the ineffectiveness of immunotherapy alone or in combination with bevacizumab in the frontline setting, the addition to a PARPi may represent a fruitful strategy to improve the outcomes of ICIs. Four phase III trials are exploring the combination of PARPis and ICIs in addition to the ChT backbone in treatment-naïve OC. The results of these trials, which are expected in the second half of 2023, will be crucial to delineate the role of immunotherapy in OC. The demonstration of a survival benefit for the combination may establish a new SoC in the treatment of advanced OC, while negative results would possibly hamper any further development of ICIs in this disease.

The PARPi–ICI combination might also be a promising approach in the context of ROC, especially in those patients who are not candidates for platinum retreatment. In this scenario, the previously mentioned NITCHE trial would provide, if positive, a ChT-free approach for patients that are traditionally offered standard single-agent ChT, with low response rates. Furthermore, the triple combination of an ICI, a PARPi, and an antiangiogenic drug seems to be very active in relapsing OC, as shown by the result of the MEDIOLA trial in which the response rate with the triplet was more than doubled compared with the doublet PARPi + ICI.

Broadening the horizon, the PARPi + ICI combination may also be exploited in other gynecological malignancies, such as TP53-mutated endometrial cancer. These tumors show molecular features that may predict the sensitivity to PARP inhibition, such as the high prevalence of HRD^71^; by contrast, the anti PD-L1 durvalumab demonstrated a meaningful activity in endometrial cancer with deficient DNA mismatch repair in the phase II PHAEDRA trial.^72^ Therefore, the combination of durvalumab with frontline ChT followed by maintenance with durvalumab + olaparib or placebo is currently being evaluated in the double-blind, randomized phase III DUO-E trial, with a design similar to that of the previously mentioned DUO-O trial.^73^ Likewise, the ongoing part 2 of the RUBY trial will investigate if the combination of dostarlimab + ChT followed by maintenance therapy with dostarlimab and niraparib is superior to the SoC in patients with recurrent or advanced endometrial cancer.^74^

Emerging preclinical and clinical data show that although ICI monotherapy exhibits limited efficacy in the treatment of OC, the combination of this with other agents could represent an interesting field of research on which to focus our attention. In addition to the intriguing data, early phase clinical data highlight that the combination of two ICIs shows significant activity compared with a single ICI^75^; furthermore, nonpharmacological approaches such as radiotherapy, which acts as an immune sensitizer by increasing antigen presentation and by inducing cell death, may intensify the response to ICI monotherapy.^76^

### Conclusions

To date, immunotherapy alone failed to demonstrate a role in OC treatment. For this reason, the research of novel association between ICIs and other agents represents, unsurprisingly, an active field of investigation.^77^ Published data, on this purpose, including both tyrosine-kinase inhibitors and anti-angiogenetic agents,^78^ have shown interesting response rates.

In the present review we focused the attention on ICI and PARPi combinations, trying to add another piece in a complex puzzle far to be concluded. While available results show that this drug association is capable of overcoming immune unresponsiveness, future results from ongoing trials will be fundamental to shed light in this setting.
